# Physical performance determinants in competitive youth swimmers: a systematic review

**DOI:** 10.1186/s13102-023-00767-4

**Published:** 2024-01-18

**Authors:** Todd Price, Giuseppe Cimadoro, Hayley S Legg

**Affiliations:** 1 Department of Sport and Exercise Sciences, St Mary’s University Twickenham, Twickenham, England; 2 School of Education and Applied Sciences, University of Gloucestershire, Gloucester, England

**Keywords:** Strength, Power, Anaerobic, Aerobic, Body composition, Water sports

## Abstract

**Background:**

Youth swimming performance is determined by several physiological, biomechanical and anthropometric characteristics. This review aimed to identify physical performance determinants of youth swimming performance, assessing strength, power, anaerobic, aerobic and body composition measures. ˙

**Methods:**

Searches were conducted in electronic databases (PubMed and Web of Science) using keywords relating to swimming and physiological measures, supplemented by citation searching of similar reviews. A total of 843 studies were identified in the initial search. The following inclusion criteria were used: participants were competitive/trained swimmers; swimming time-trial or event was conducted; data was provided on one or more physiological parameters; study was published in English and peer-reviewed. A total of 43 studies met the inclusion criteria. Risk of bias was assessed using Joanna Briggs Institute (JBI) checklist.

**Results:**

Cross-sectional studies scored between 4–8 and randomised-controlled trials scored 8–9 on their respective JBI checklists. Youth swimming performance was determined by muscle strength, muscle power, lean body mass, anaerobic and aerobic metabolism measures in most studies, where improved performance values of these variables were conducive to swimming performance. Body fat percentage did not have a clear relationship in youth swimming performance.

**Conclusions:**

Findings of this review suggest that greater levels of muscle strength, muscle power and lean body mass are favourable in swimming performance, with muscle strength and muscle power particularly beneficial for start and turn performance. Anaerobic and aerobic metabolism measures were good determinants of swimming performance, with middle- and long-distance events more influenced by the latter. Body fat percentage has a nuanced relationship with swimming performance, where further investigation is required. Findings were inconsistent across studies, potentially due to unidentified confounding factors.

**Key points:**

• Greater muscular strength and power qualities, anaerobic and aerobic capacities, and lean body mass are conducive to swimming performance.

• Body fat percentage has a nuanced relationship with swimming performance.

• Practitioners should consider general strength and power training as a useful tool to enhance performance in their youth competitors.

## Background

In order to develop physical qualities to enhance performance, it is important for the training practitioner to have an understanding of the determinants that impact the performance via the dynamic correspondence approach [87]. Research identifying performance predicting factors has been conducted in many sports, including cycling [7], football [75], rowing [65], rugby [6], running [84], weightlifting [32], swimming [67], volleyball [63] and triathlon [81]. In swimming, physiological variables impacting performance have been investigated in multiple studies for both young [4, 8, 25, 33, 35] and adult swimmers [67, 68, 79, 90].

The physical assessment of youth athletes can be a beneficial and worthwhile undertaking. Assessments can be utilised to identify strengths and weaknesses, evaluate the effectiveness of training programs, providing metrics to identify targets and assist in talent identification and selection [89]. Multiple variables can be measured through physical testing of youth athletes, fundamentally, swimming performance is determined by a combination of anthropometric, biomechanical, physiological, psychological and technical factors [35].

Strength training is common in youth sports programs [19] and has shown relationships with sports performance [18]. In shorter swimming events, success is dependent on application of force through water [3, 30], alongside high requirements of strength and power [57]. The contribution of all three energy systems in sprint swimming are used to varying degrees: Phosphagen (5–80%), Glycolytic (2–80%), Aerobic (2–54%) [73]. The dominance of anaerobic processes and high force output required in sprint swimming provides reasoning for the popularity of strength training outside of the pool. Consequently, common dryland exercises found in strength and conditioning programs for swimmers have been studied and used to predict swimming performance [57, 59]. Frequently, strength and power exercises involving generating force through the upper limbs have shown relationships with swimming performance in youth and senior swimmers [11, 24, 37, 58, 66]. These studies used exercises such as the bench press, pull up, lat pull down, and movements that engage the latissimus dorsi, pectorals and triceps, all of which are dominant muscles activated in the arm action during freestyle swimming [66].

In endurance-based swimming events, energetic contributions are 0–30% phosphagen, 10–65% glycolytic and 5–90% aerobic [73]. In the 400 m freestyle, 79% of variance in performance can be determined by swimming velocity at 85% of V̇O_2_max or 4 mmol/L blood lactate (BL) concentration [72]. Other studies in senior swimmers have also demonstrated relationships between V̇O_2_max [20] and BL [27] with swimming performance, indicating success in endurance swimming events depends on velocity relationships with these variables.

Across all distances of swimming events, somatic markers such as body fat percentage (BF%) [15] and lean body mass (LBM) [78] have shown relationships with youth swimming performance. Lower BF% has been shown to reduce drag in the water [40]. LBM may influence swimming performance due to its relationship with strength and power measures [45], as strength and power have been shown to predict swim performance [38, 39].

It is clear a combination of aerobic and anaerobic capacity, strength, power and anthropometric parameters play an important role in swimming performance. Subsequently, enhanced swim performance has been demonstrated in youth swimmers throughout various studies, that use a range of variables, both anthropometric and capacity based including upper extremity length, leg power and handgrip strength [25], stroke index, arm span and V̇O_2_peak [35], sitting height, aerobic speed and endurance, and swimming index [76].

Evidence suggests the development and growth of adolescents has an impact on physical capacity and skill acquisition [48], meaning determinants of swimming performance could differ in comparison to adults. Furthermore, research has shown training time spent on speed, power, endurance, technique and dryland varies in youth, adult and masters swimmers, where dryland training time was highest in varsity and international level swimmers [88]. These findings may be considered an observation of physical qualities that coaches perceive important at different ages. Therefore, studying physiological indicators of swim performance in adolescents is useful in providing specific measures for this demographic group.

In the current literature there are some reviews that help us to understand youth swimming performance, but none that specifically comment on the relationships between dryland exercises and assessments with swimming performance. This review aims to scrutinise the youth swimming performance athletic determinants paving the way for future research to explore how youth swimming training can be optimised and providing clear and updated guidelines for coaches and swimmers.

## Methods

### Literature search strategy

**Fig. 1:**
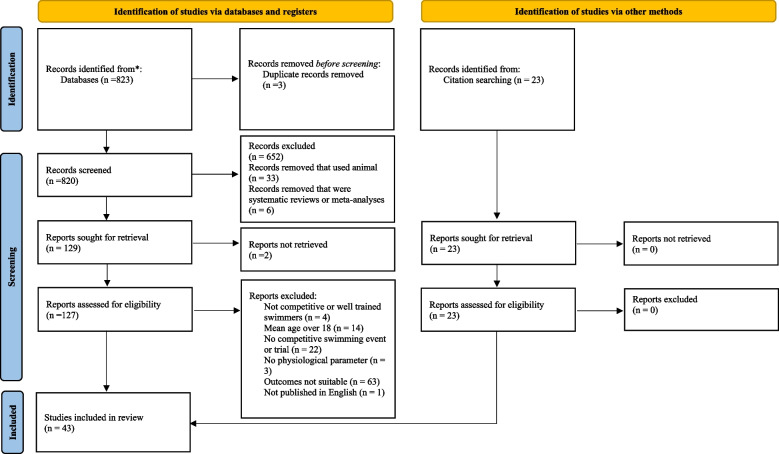
Search, screening and selection process

For this review, the PRISMA statement was used as a guideline for the procedures described in this section [54]. Searches were conducted on electronic databases which included PubMed and Web of Science using the following terms and Boolean operators: “swim*” AND “youth” AND “determinants” OR “indicators” OR “predictors” AND “performance” AND “physiological” OR “strength” OR “power” OR “aerobic” OR “anaerobic” OR “endurance” OR “body composition”. All searches were constrained to articles that were published in English and from the date of the first record to 4^th^ April 2023 A visual overview of the study selection process is displayed in Fig. 1.

### Inclusion and exclusion criteria

In order to identify eligible studies, an inclusion criterion was applied to the screened papers. Studies were included if participants were competitive or trained pool swimmers, a swimming time-trial or event was conducted and provided data on one or more of the following parameters: V̇O_2_, BL, power measures (e.g., peak power in countermovement jump) strength measures (e.g., peak force in isokinetic shoulder flexion) and body composition (e.g. body fat percentage). Furthermore, the paper must have been published in English within a peer-reviewed journal.

Non-eligible studies included papers that used participants who were non-swimmers (e.g., untrained/ less than 6-months swimming experience), did not compete in competitive swimming (e.g., triathlon, water polo, synchronized swimming), were part of another population group (e.g., Paralympic), mean age was over 18 years, or were in poor health/injured. Additionally, review articles (qualitative review, systematic review and meta-analysis) were not included. Finally, any articles that did not present a complete description of their methods and/or results were omitted.

### Study selection

All search results were imported into a citation software for the screening process. The initial search yielded 823 publications. An additional manual search was conducted via reference lists of previous reviews similar to this topic, where 23 potential studies were identified. Once duplicates (*n* = 3) were removed, the remaining studies (*n* = 843) titles and abstracts were screened for eligibility by two reviewers, leaving 152 relevant papers to be considered for this review. The article full text was not available in two studies, leaving 150 available to be assessed by the same two reviewers. These studies were judged for suitability, resulting in a further 107 being disregarded for the subsequent reasons: participants were not competitive swimmers (*n* = 4), participants mean age was over 18 years old (*n* = 14), no swimming event or trial was conducted (*n* = 22), no physiological parameter was measured (*n* = 3), study outcomes were not suitable (*n* = 63), study was not published in English (*n* = 1). This resulted in 43 papers being selected for the review.

### Analysis of results

The relationships found in the reviewed studies were identified as either weak (0.10–0.39), moderate (0.40–0.69), strong (0.70–0.89) or very strong (0.90–1.00) [77]. For assessing the quality of research, JBI critical appraisal tools for cross-sectional studies and randomised-controlled trials were used as they are recommended tools for conducting systematic reviews [47]. This process involves scrutinising the methodology of each study against eight (cross-sectional studies) or thirteen (randomized-controlled trials) points of scientific rigor, assessing quality and addressing potential bias in design, conduct and analysis. Consequently, each study is awarded a score from 0–8 or 0–13 respectively, where a higher score equals a better quality study. Studies were not removed based on their rating, the purpose of the appraisal was to provide a grading of study quality for the studies used in this review.

## Results

### Participant characteristics

Participant characteristics and JBI scores across the 43 eligible studies are summarised in Table 1. A total of 1837 participants are included in this review, where mean ages ranged between 10.3 ± 1.0 and 17.5 ± 3.5. The competition level of participants was reported in all but 13 studies who either stated [9, 16, 21, 50, 70–72] or did not state the participants were competitive [29, 35, 42, 43, 52, 60]. For studies who stated competitive level, one included only county level participants [41], one included only regional level participants [62], thirteen included only national level participants [1, 2, 5, 12, 15, 17, 44, 53, 61, 74, 76, 81, 86] and one included only international participants [23]. Twelve studies recruited a combination of participants who were competing at either national or regional level [13, 25, 26, 36, 38, 39, 51, 55, 56, 78, 82, 83], one recruited international and national level participants [58] and one included regional, national and international participants [31].

**Table 1:** Participant characteristics and JBI scores

**Author**	***n***		**Gender**	**Age (years)**		**Height (cm)**	**Body mass (kg)**	**Maturity Assessment**	**Training experience (years)**	**Competition level**	**JBI Score**
Almeida et al. 2020 [1]	28		Male = 14, Female = 14	Male = 16.6 ± 1.8, Female = 15.6 ± 2.6		Male = 178.5 ± 8.1, Female = 163.4 ± 6.7	Male = 70.5 ± 9.9, Female = 56.0 ± 6.7	No	Well trained	National	5
Amara et al. 2021 [2]	33		Male	16.46 ± 0.59		180.56 ± 5.69	72.82 ± 8.41	No	9.50 ± 0.71	National	7
Bond et al. 2015 [5]	50		Male = 21, Female = 29	13.6 ± 1.7		164 ± 1.5	54.4 ± 1.4	No	Not stated	National	5
Chatard et al. 1990 [9]	84		Male = 48, Female = 36	Male = 16.1 ± 3.5, Female = 14.6 ± 2		Male = 170 ± 12, Female = 165 ± 8	Male = 60 ± 14, Female = 55 ± 9	No	Not stated	Competitive, level not stated	7
de Barros Sousa et al. 2017 [12]	12		Male = 6, Female = 6	15.7 ± 1.1		173.3 ± 9.5	66.1 ± 9.5	No	Not stated	National	5
de Mello Vitor & Böhme, 2010 [13]	24		Male	13.0 ± 0.7		163.5 ± 6.3	52.3 ± 8.4	Yes	3–4 years	Regional or national	7
Dos Santos et al. 2021 [15]	85		Male = 50, Female = 35	Male = 13.56 ± 1.80, Female = 12.60 ± 1.88		Male = 165.07 ± 11.06, Female = 154.68 ± 8.82	Male = 54.72 ± 10.08, Female = 47.63 ± 10.88	Yes	Not stated	National	5
Duché et al. 1993 [16]	25		Male	11.3 ± 1		147.3 ± 9.7	38.3 ± 7.8	Yes	Not stated	2 years competitive experience, level not stated	7
**Author**	***n***		**Gender**	**Age (years)**		**Height (cm)**	**Weight (kg)**	**Maturity Assessment**	**Training experience (years)**	**Competition level**	**JBI Score**
Espada et al. 2015 [17]	12		Male	16 ± 3.2		175.2 ± 9.1	65.4 ± 8.9	No	6 years	National	5
Ferreira et al. 2021 [21]	34		Male = 24, Female = 10	12.07 ± 1.14		155 ± 10	45.42 ± 9.22	Yes	Not stated	More than 1 year of competitive experience, level not stated	8
García-Ramos et al. 2016 [23]	20		Female	15.3 ± 1.6		166.9 ± 5.9	57.2 ± 7.4	No	Not stated	International	5
Geladas et al. 2005 [25]	263		Male = 178, Female = 85	12.78 ± 0.047		Male = 165.5 ± 0.7, Female = 161.2 ± 0.6	Male = 54.1 ± 0.7, Female = 48.3 ± 0.6	Yes	Not stated	Regional or national	7
Girold et al. 2006 [26]	37		Male = 16, Female = 21	17.5 ± 3.5		173 ± 14	63 ± 14	No	Not stated	Regional or national	8
Hawley et al. 1992 [29]	30		Male = 12, Female = 10	Male = 13.6 ± 1.3, Female = 13.2 ± 1.9		Not stated	Male = 54.4 ± 7.6, Female = 56.2 ± 10.1	No	At least 3 months prior to study	Not stated	6
Hellard et al. 2018 [31]	26		Not stated	16 ± 1		178 ± 8	65 ± 8	No	Not stated	Regional, national and international	4
Jürimäe et al. 2007 [35]	29		Not stated	13.0 ± 1.8		163.6 ± 11.9	51.6 ± 13.0	Yes	3.0 ± 1.1 years	Not stated	7
**Author**	***n***		**Gender**	**Age (years)**		**Height (cm)**	**Weight (kg)**	**Maturity Assessment**	**Training experience (years)**	**Competition level**	**JBI Score**
Kalva-Filho et al. 2018 [36]	25		Male = 14, Female = 11	15 ± 1, 13 ± 1		165 ± 8	59.3 ± 8.2	No	5 years minimum	Regional or national	6
Keiner et al. 2015 [39]	14		Male	17.5 ± 1.6		1.81 ± 0.0	70.21 ± 4.88	No	7 years minimum	Regional or national	6
Keiner et al. 2019 [38]	21		Male = 12, Female = 9	17.5 ± 2		177.3 ± 10.1	69.5 ± 11.4	No	Not stated	Regional or national	5
Klika & Thorland, 1994 [41]	12		Male	10.3 ± 1.0		Faster swimmers = 1.412 ± 0.104, Slower swimmers = 1.406 ± 0.062	Faster swimmers = 35.1 ± 11.0, Slower swimmers = 30.2 ± 3.0	No	Not stated	Competitive—local/age-group competitions	7
Lätt et al. 2009 [42]	26		Female	12.7 ± 2.2 to 14.6 ± 1.9 (conducted over 3 years)		160.9 ± 9.3	50.3 ± 9.2 to 55.8 ± 8.8	Yes	3.7 ± 1.8 years	Not stated	7
Lätt et al. 2010 [43]	25		Male	15.2 ± 1.9		176 ± 0.09	63.3 ± 10.9	Yes	5.6 ± 1.5 years	Not stated	7
Loturco et al. 2015 [44]	10		Male	17.0 ± 0.7		177 ± 0.05	70.4 ± 6.3	No	Well trained	National	4
Maszczyk et al. 2012 [50]	189		Male	12 ± 0.5		Not stated	Not stated	No	4 years	Competitive, level not stated	6
**Author**	***n***		**Gender**	**Age (years)**		**Height (cm)**	**Weight (kg)**	**Maturity Assessment**	**Training experience (years)**	**Competition level**	**JBI Score**
Mezzaroba et al. 2013a [51]	71		Male = 41, Female = 30	M1 = 13.6 ± 2.1, M2 = 14.3 ± 1.9, F1 = 13.3 ± 2.0, F2 = 13.3 ± 2.6		M1 = 165 ± 14, M2 = 166 ± 10, F1 = 158 ± 9, F2 = 157 ± 10	M1 = 56.6 ± 16.4, M2 = 55.1 ± 12.1, F1 = 51.3 ± 9.3, F2 = 52.1 ± 13.5	Yes	2 years minimum	Regional or national	5
Mezzaroba et al. 2013b [52]	33		Male = 17, Female = 16	Male = 13.559 ± 2.346, Female = 13.179 ± 2.255		Male = 161.120 ± 11.806, Female = 154.930 ± 8.986	Male = 53.947 ± 13.616, Female = 48.707 ± 8.662	Yes	2 years minimum	Not stated	5
Mitchell et al. 2018 [53]	48		Male = 22, Female = 26	Male = 16.5 ± 1.2, Female = 15.5 ± 1.1		100 m group—177.9, 200 m group—176.9	Measured but not stated	Yes	Not stated	National	6
Morais et al. 2016a [55]	100		Male = 49, Female = 51	12.3 ± 0.74		158.9 ± 7.94	48.8 ± 8.29	Yes	3.1 ± 0.71 years	Regional or national	7
Morais et al. 2016b [56]	27		Male = 12, Female = 15	Male = 13.55 ± 0.72, Female = 13.16 ± 0.93		C1 = 168.57 ± 7.61, C2 = 160.44 ± 7.32, C3 = 159.36 ± 5.80	C1 = 58.37 ± 4.86, C2 = 50.92 ± 5.96, C3 = 50.43 ± 8.34	Yes	3.67 ± 0.73 years	Regional or national	7
Morouço et al. 2014 [58]	34		Male	17.2 ± 2.72		1.76 ± 0.09	67.4 ± 9.94	No	5 years minimum	National and international	4
Papoti et al. 2009 [61]	25		Male = 15, Female = 10	16 ± 3		168 ± 5.04	63 ± 6.07	No	5 years minimum	National	5
Papoti et al. 2013 [60]	12		Male = 9, Female = 3	Male = 16 ± 1.0, Female = 15 ± 0.6,		Male = 1.69 ± 0.1, Female = 157 ± 0.1	Male = 60 ± 3.0, Female = 55 ± 3.9	No	Trained	200 m freestyle time = 71% of world record, level not stated	7
**Author**	***n***	**Gender**		**Age (years)**		**Height (cm)**	**Weight (kg)**	**Maturity Assessment**	**Training experience (years)**	**Competition level**	**JBI Score**
Pardos-Mainer et al. 2015 [62]	67	Male = 38, Female = 29		14.3 ± 2.2		164.2 ± 12.1	55.6 ± 13.2	Yes	9.7 ± 2.3 years	Regional	8
Potdevin et al. 2011 [70]	23	Male and Female		Interventio*n* = 14.1 ± 0.2, Control = 14.3 ± 0.2		Intervention = 161 ± 12, Control = 158 ± 12	Intervention = 50.03 ± 9.04, Control = 50.85 ± 12.71	Yes	Not stated	Competitive experience. Intervention = 3.5 ± 1.3, Control = 3.1 ± 1.9, level not stated	9
Reis et al. 2010 [71]	22	Male		16.9 ± 2.6		184 ± 6	69.1 ± 7.9	No	G1 = 10.2 ± 1.6, G2 = 11.0 ± 2.5 years	Competitive, level not stated	5
Ribeiro et al. 1990 [72]	15	Not stated		16 ± 2		171 ± 4	63 ± 8	No	Not stated	Competitive, level not stated	6
Rodríguez et al. 2015 [74]	36	Male = 26, Female = 10		Male = 15.5 ± 2.2, Female = 15.4 ± 1.8		Male = 176.2 ± 9.2, Female = 169.9 ± 5.3	Male = 63.3 ± 10.7, Female = 57.8 ± 5.7	No	5.6 ± 1.5 years	National	7
Saavedra et al. 2010 [76]	133	Male = 66, Female = 67		Male = 13.60 ± 0.56, Female = 11.51 ± 0.55		Male = 171.12 ± 7.50, Female = 154.75 ± 7.47	Male = 57.95 ± 8.18, Female = 43.96 ± 7.17	Yes	Not stated	National	7
**Author**	***n***		**Gender**	**Age (years)**	**Height (cm)**		**Weight (kg)**	**Maturity Assessment**	**Training experience (years)**	**Competition level**	**JBI Score**
Seffrin et al. 2021 [78]	49		Male = 33, Female = 16	Male = 11.50 ± 0.52, Female = 11.67 ± 0.52, Male = 13.53 ± 0.62, Female = 13.33 ± 0.52, Female = 16.25 ± 0.96	Male = 151.70 ± 8.46, Female = 155.55 ± 4.58, Male = 164.74 ± 6.94, Female = 160.48 ± 4.53, Female = 159.93 ± 5.59		Male = 42.08 ± 8.60, Female = 47.07 ± 9.76, Male = 53.19 ± 10.42, Female = 50.15 ± 5.41, Female = 59.53 ± 6.59	No	Not stated	Regional or national	8
Smith et al. 1988 [81]	16		Male	17.4 ± 2.3	179.8 ± 6.4		71.4 ± 7.1	No	Not stated	National	7
Strzala et al. 2015 [82]	27		Male	15.7 ± 1.98	180 ± 2		70.03 ± 9.35	No	Not stated	Regional or national	7
Strzała et al. 2019 [83]	15		Male	17.3 ± 0.59	181.1 ± 6.25		74.8 ± 4.40	No	Not stated	Regional or national	8
Unnithan et al. 2009 [86]	10		Female	15.3 ± 1.5	166.3 ± 6 cm		48.7 ± 3.2	Yes	6.4 ± 1.9 years	National	4

### Study design and JBI Scores

Over the 43 studies, swimming velocity, swimming trials, personal best times, LEN Ligue (Européenne de Natation)/FINA points were used as the swimming performance parameters. LEN/FINA points are calculated by relating personal best times to current world records via mathematical equation [22].

Across the 43 studies, a total of 18 measured strength and power variables, with three studies measuring only strength [2, 26, 58], five measuring only power [41, 53, 55, 56, 70] and ten measuring at least one variable of each [23, 25, 38, 39, 44, 50, 62, 67, 76, 80]. One study directly measured the propulsion force of the arms during swimming as the strength and power test [15]. Three of the 18 studies investigated the influence of strength and power variables in relation to swimming start and/or turn performance [23, 38, 39], the remainder of studies researched strength and power variables with swimming performance alone.

Energetic measures were explored relative to swimming performance in 29 papers in this review. Studies reported BL values [12, 36, 51, 52, 58], measures of V̇O_2_ [9, 41, 62, 81, 82] or BL and V̇O_2_ [1, 17, 21, 31, 35, 38, 39, 61, 67, 71, 72, 74, 86], with one study measuring V̇O_2_ and anaerobic power [16] and one measuring anaerobic power alone [29]. Investigations also operated test measurements representing energetic capacities including critical speed [13, 52, 53], lung capacity [50] and a shuttle run endurance stage test [76].

A measurement of body composition in relation to swimming performance was incorporated into the design of 18 studies included in this review. Ten studies reported only a measure of body fat [5, 13, 15, 16, 25, 41, 51, 53, 71, 76], three only LBM or fat free mass [38, 82, 83] and four reported both [39, 52, 62, 78]. Methods of obtaining these measures included bioelectrical impedance [62], densitometry [38, 39, 41], absorptiometry [52, 78] and skin folds [5, 13, 15, 16, 25, 51, 53, 76, 82, 83].

A total of 14 studies stratified their sample, two by grade of performance [5, 55], two by age [78, 83], one by age and performance [41], eight by gender [9, 12, 25, 29, 51, 52, 62] and two by gender and performance [53, 76]. The remaining studies did not stratify their samples. Seventeen studies conducted a maturity assessment amongst their participants [13, 15, 16, 21, 25, 35, 38, 39, 51–53, 55, 56, 62, 70, 76, 86].

Of the studies included in this review, 95.35% (41) were cross-sectional and 4.65% (2) were randomised-controlled trials. All cross-sectional studies scored 4, 5, 6, 7 or 8 and randomised-controlled trials scored 8 or 9 on their respective JBI checklists. 81.4% of cross-sectional studies had points deducted for failing to describe inclusion criteria. Differences in JBI scores were due to investigations not describing participants in detail, failing to identify confounding factors, not providing strategies to deal with confounding factors and not using appropriate statistical analysis. For randomised controlled trials, each study had points deducted for items relating to blinding of participants, treatment and assessors. These factors are challenging to control in training intervention studies.

### Maximal strength and explosive power measures

Evidence for greater strength and/or power being a contributing factor for better swim performance was found in 18 studies, whether via simple correlation or multiple regression analysis (Table 2). A mixture of isokinetic and multi joint actions were used to measure strength and power across the included studies.

**Table 2:** Summary of maximal strength and explosive power relevant measures and major findings of the reviewed studies

Author	Relevant Measures	Major Findings
Amara et al. 2021 [2]	1RM push-up	1RM push-up significant negative correlation with 25 and 50 m front crawl (*r* = − 0.968, *r* = − 0.955), and the 25 or 50 m front crawl with arms (*r* = − 0.955, *r* = -0.941)
Dos Santos et al. 2021 [15]	PFA	PFA amongst best predictors of 50 m freestyle performance in backwards regression analysis greater PFA were conducive to performance
García-Ramos et al. 2016 [23]	SJ, CMJ, isometric strength	TOV and PP normalised to BW of SJ negatively correlated with time to 5 m (*r* = -0.56; *p* < 0.05, *r* = -0.57; *p* < 0.01). TOV and PP normalised to BW of CMJ negatively correlated with time to 5 m (*r* = -0.62; *p* < 0.01, *r* = -0.61; *p* < 0.01) and time to 10 m (*r* = -0.49; *p* < 0.05, *r* = -0.55; *p* < 0.05) Loaded SJ PP normalised to BW negatively correlated with time to 5 m, 10 m and 15 m at; 25%BW (*r* = -0.62; *p* < 0.01, *r* = -0.55; *p* < 0.05, *r* = -0.57; *p* < 0.01), 50%BW (*r* = -0.63; *p* < 0.01, *r* = -0.51; *p* < 0.05, *r* = -0.54; *p* < 0.05), 75%BW (*r* = -0.57; *p* < 0.01, *r* = -0.54; *p* < 0.05, *r* = -0.64; *p* < 0.01), 100%BW (*r* = -0.54; *p* < 0.05, *r* = -0.47; *p* < 0.05, *r* = -0.64, *p* < 0.01). Loaded SJ PV negatively correlated with time to 5 m, 10 m and 15 m at; 25%BW (*r* = -0.66; *p* < 0.01, *r* = -0.57; *p* < 0.01, *r* = -0.63; *p* < 0.01), 50%BW (*r* = -0.72; *p* < 0.01, *r* = -0.57; *p* < 0.01, *r* = -0.63, *p* < 0.01), 75%BW (*r* = -0.63; *p* < 0.01, *r* = -0.59; *p* < 0.01, *r* = -0.68; *p* < 0.01), 100%BW (*r* = -0.57; *p* < 0.05, *r* = -0.50; *p* < 0.05, *r* = -0.64; *p* < 0.01). No significant correlations between isometric strength measures and swimming start performance
Geladas et al. 2005 [25]	HJ, HGS	Negative correlations between HJ and 100 m time in boys (*r* = -0.58, *p* < 0.01) and girls (*r* = -0.25, *p* < 0.01). Negative correlation between HGS and 100 m time in boys (*r* = -0.73, *p* < 0.01), not girls
Girold et al. 2006 [26]	Isometric and concentric strength	100 m freestyle performance in competition positively correlated to the strength of the elbow flexors and extensors under isometric (*r* = 0.57; 0.54; *p* < 0.05) and concentric conditions (*r* = 0.64 to 0.67; 0.66; *p* < 0.05)
Keiner et al. 2015 [39]	1RM squat, 1RM bench press, 1RM sit-up, 1RM bent over row, 1RM deadlift, SJ, CMJ	15 m freestyle negatively correlated with 1RM squat, SJ, CMJ, 1RM bench press, 1RM bent over row, 1RM deadlift and 1RM sit-up (*r* = -0.76; -0.94; -0.92; -0.84; 0.81; -0.68; -0.51; *p* < 0.05). 25 m freestyle negatively correlated with 1RM squat, SJ, CMJ, 1RM bench press, 1RM bent over row and 1RM deadlift (*r* = -0.75; -0.94; -0.91; -0.85; -0.83; -0.68; *p* < 0.05). 50 m freestyle negatively correlated with 1RM squat, SJ, CMJ, 1RM bench press, 1RM bent over row, 1RM deadlift and 1RM sit-up (*r* = -0.72; -0.82; -0.82; -0.83; -0.80; -0.68; -0.48; *p* < 0.05). 100 m freestyle negatively correlated with 1RM squat, SJ, CMJ, 1RM bench press, 1RM bent over row, 1RM deadlift and 1RM sit-up (*r* = -0.68; -0.77; -0.77; -0.81; -0.77; -0.64; -0.38; *p* < 0.05). 50 m breaststroke negatively correlated with 1RM squat, SJ, CMJ, 1RM bench press, 1RM bent over row, 1RM deadlift and 1RM sit-up (*r* = -0.70; -0.87; -0.85; -0.79; -0.78; -0.65; -0.39; *p* < 0.05). 100 m breaststroke negatively correlated with 1RM squat, SJ, CMJ, 1RM bench press, 1RM bent over row, 1RM deadlift and 1RM sit-up (*r* = -0.73; -0.86; -0.84; -0.82; -0.80; -0.67; -0.38; *p* < 0.05). 50 m backstroke negatively correlated with 1RM squat, SJ, CMJ, 1RM bench press, 1RM bent over row, 1RM deadlift and 1RM sit-up (*r* = -0.54; -0.53; -0.53; -0.65; -0.65; -0.51; -0.31; *p* < 0.05) 100 m backstroke negatively correlated with 1RM squat, SJ, CMJ, 1RM bench press and 1RM bent over row (*r* = -0.33; -0.36; -0.37; -0.37; -0.39; *p* < 0.05)
Keiner et al. 2019 [38]	SJ, CMJ, 1RM squat, 1RM bench press	1RM bench press and squat combined positively correlated with 50 m freestyle performance (R2 = 0.62), 100 m freestyle performance (R2 = 0.45), swimming power (R2 = 0.65) and 15 m start performance (R2 = 0.50). Start performance to 15 m negatively correlated with 1RM squat (r = -0.67; *p* < 0.05), SJ (*r* = -0.78; *p* < 0.05) and CMJ (*r* = -0.77; *p* < 0.05). Start performance to 5 m negatively correlated with 1RM squat (*r* = -0.65; *p* < 0.05) and SJ (*r* = -0.56; *p* < 0.05). Turn performance to 5 m negatively correlated with SJ (*r* = -0.65; *p* < 0.05) and CMJ (*r* = -0.75; *p* < 0.05)
Klika & Thorland, 1994 [41]	FM, LBM, vertical jump, peak VO_2_, arm-stroke force and leg stroke force	Multiple discriminant function identified leg kick force, peak VO_2_, stroke efficiency and muscularity as predictors of performance level (Multiple discriminant function coefficient = unstandardized 0.822, 0.221, 0.732; standardized 1.87, 1.48, 1.08). Vertical jump power showed no relationship with performance
Loturco et al. 2015 [44]	PF, MPP, IMP, isometric strength, SJ, CMJ	Tethered swimming PF, AF, RFD and IMP negatively correlated with 50 m freestyle time (*r* = -0.82; -0.85; -0.72; -0.76; *p* < 0.01). Tethered swimming PF and AF negatively correlated with 100 m freestyle time (*r* = -0.74; -0.67; *p* < 0.05). Negative correlation between 50 m swimming time and JS MPP (*r* = -0.70, *p* < 0.05). Correlations between isometric BP and QS (PF and RFD) were not significant
Maszczyk et al. 2012 [50]	HJ	HJ key predictive factor of 50 m freestyle performance in when used in regression models
Mitchell et al. 2018 [53]	CMJ	Negative correlations between swimming performance improvements and loaded CMJ height in 100 m males (*r* = -0.79), 200 m males (*r* = -0.47) and 100 m females (*r* = -0.39) through multiple linear regression model
Morais et al. 2016a [55]	Medicine ball TV, SJ and CMJ height	Cluster 1 (talented, fastest swimmers) characterized as having a high SJ (0.34 m ± 0.06 vs 0.24 m ± 0.03, F = 11.18, *p* < 0.001) and (TV) (7.58 ± 0.28 vs 6.07 ± 0.81 ms, F = 8.18, *p* = 0.002) compared to cluster 3 (slowest swimmers)
Morais et al. 2016b [56]	Medicine ball TV	TV negatively correlated with 100 m freestyle time (*r* = -0.42; *p* = 0.03). Regression model identified TV influences power to overcome drag, which in turn influences both swimming velocity and propelling efficiency, explaining 69% of variance in performance
Pardos-Mainer et al. 2015 [62]	HGS, HJ, isometric strength, 30 m running sprint	HJ, 30 m sprint velocity, HGS, isometric crawl force, knee extension isometric force all had significant correlations with 50 m freestyle time (*r* = -0.561; 0.538; -0.511; -0.269; -0.267; *p* < 0.05)
Potdevin et al. 2011 [70]	CMJ, SJ	6 weeks of plyometric training improved maximal glide speed (2.28 ± 0.19 vs. 2.41 ± 0.27 ms, *p* < 0.05, ES = 0.26), 400 m freestyle velocity (0.96 ± 0.09 vs. 0.92 ± 0.10 ms, ES = 0.15; *p* < 0.05) and 50 m freestyle velocity (1.29 ± 0.15 ms vs. 1.25 ± 0.18 ms, ES = 0.1, *p* < 0.05). Positive correlation between change in SJ height and change in 50 m freestyle velocity (*r* = 0.73, *P* < 0.05)
Saavedra et al. 2010 [76]	HJ, HGS, trunk power, isometric strength,	Positive correlations between swimming performance and HJ (*r* = 0.312 p ≤ 0.05), HGS (*r* = 0.508; p ≤ 0.05), abdominals in 30 s (*r* = 0.346; p ≤ 0.05), flexed arm hang (*r* = 0.351; p ≤ 0.05)
Seffrin et al. 2021 [78]	HGS, SJ, CMJ, isokinetic strength	HGS negatively correlated with 100 m freestyle performance in males and females (*r* = -0.77; -0.74; p ≤ 0.05) and 400 m freestyle performance in males (*r* = -0.67; p ≤ 0,05). CMJ negatively correlated with 100 m and 400 m freestyle performance in males (*r* = -0.65; -0.55; p ≤ 0.05). Flexion and extension torque and power of the upper and lower limbs negatively correlated with 100 m (*r* = -0.84 to -0.51; p ≤ 0.05) and 400 m freestyle performance (*r* = -0.59 to -0.51; p ≤ 0.05)
Strzała et al. 2019 [83]	CMJ, isometric strength	CMJ performance (cm and J) positively correlated with front crawl velocity (*r* = 0.57; 0.69; *p* < 0.05). Positive correlations between knee flexion, knee extension and freestyle velocity (*r* = 0.56; 0.57; *p* < 0.05)

Multi-joint exercises were used in five studies, where 1-repetition maximum tests (1RM) were used by Amara et al. [2], Keiner et al. [38] and Keiner et al. [39]. Significant relationships between 1RM, swimming [2, 28, 39] and start performance [38, 39] were reported, where greater 1RM scores were associated with superior performance. 1RM push-up was associated with faster times in the 25 and 50 m front crawl and front crawl arms only [2]. Keiner et al. [38] reported moderate correlations between 15 m, 50 m and 100 m freestyle with bench press and squat 1RM when combined in a multiple regression analysis, where higher 1RM scores were conducive to swim performance. Strong correlations were found with 5 m and 15 m start performance with 1RM squat scores alone, where stronger squatters had faster start times. Similarly, Keiner et al. [39] demonstrated higher 1RM scores were associated with faster swim times over multiple sprint distances (15-100 m) across freestyle, breaststroke and backstroke, where weak to very strong correlations with 1RM squat, bench press, bent over row, deadlift and sit-up. A sit-up test was used in another study, but was maximal repetition rather than 1RM, where a weak correlation was found between abdominal power and swim performance [76]. Loturco et al. [44] used isometric quarter-squat and bench press as their strength tests, but no significant correlations were found with 50 m and 100 m freestyle performance.

In the eight studies that used isokinetic dynamometer devices to evaluate muscle strength and power, all but one found significant relationships with swim performance [23]. This study investigated swimming start performance with isometric flexion and extension measures of the knee, where no significant correlations were found. Similar isometric measures of the knee were conducted in three other studies but were compared to freestyle swimming velocity [82], 50 m freestyle time [62] and 100 m and 400 m freestyle performance [78]. Weak to strong correlations were found between knee flexion and extension with freestyle velocity over 50 m [82], isometric knee extension force and 50 m freestyle time [62] and knee flexion and extension torque and power with 100 m and 400 m freestyle performance [78]. Two studies investigated relationships between isometric force of the shoulder and freestyle performance over various distances. Isometric shoulder flexion measures had weak correlations with 50 m freestyle time [62] and shoulder internal and external rotation presented moderate to strong correlations with 100 m and 400 m times [78]. Upper limb strength and power was also measured by Girold et al. [26] where flexion and extension measures of the elbow showed moderate to strong correlations with 100 m freestyle performance under isometric and concentric conditions. One study measured the propulsion force of the arms during 30 s maximal freestyle efforts using a dynamometer. This measurement was considered a key predictor of 50 m freestyle performance in this study when used in an allometric approach alongside other variables [15]. Handgrip strength displayed moderate to strong correlations with swimming performance or velocity in three studies for males [25, 62, 78] and one in both males and females [77].

Jump performance was assessed in 14 studies, where tests including countermovement jumps (CMJ), squat jumps (SJ) and broad/horizontal jumps (HJ) were used. Weak to very strong correlations were found between CMJ, SJ and HJ measures with start performance [23, 38, 39] and swim performance [25, 39, 44, 50, 53, 62, 70, 76, 78, 83]. One study found no relationship between vertical jump and swim performance, but the type of jump was not stated [41]. Morais et al. [55] conducted a cluster analysis between their participants, finding SJ (0.34 m ± 0.06 vs 0.24 m ± 0.03, F = 11.18, *p* < 0.001) and CMJ (0.36 m ± 0.05 vs 0.26 m ± 0.03, F = 11.16, *p* < 0.001) score discriminated the talented, faster swimmers from the non-proficient swimmers, respectively. Turn performance was analysed in one study, revealing SJ and CMJ had strong correlations with turn performance to 5 m [38]. Potdevin et al. [70] conducted a maximal glide test, where scores improved after 6 weeks of plyometric training (2.28 ms ± 0.19 vs. 2.41 ms ± 0.27, *p* < 0.05, ES = 0.26). Alongside jump measures, Morais et al. [56] found a moderate correlation between medicine ball throwing velocity and 100 m freestyle performance and Morias et al. [55] characterised faster, talented swimmers as having higher medicine ball throwing velocity compared non-proficient swimmers (7.58 ± 0.28 vs. 6.07 ± 0.81 ms, F = 8.18, *p* = 0.002).

### Anaerobic and aerobic measures

Testing related to anaerobic and aerobic measures occurred in 30 studies, all of which found at least one relationship between an anaerobic and/or aerobic variable and swim performance (Table 3). Assessment of anaerobic and aerobic profiles of participants was commonly through BL, V̇O_2_ measures, force, power and velocity profiles.

**Table 3:** Summary of anaerobic and aerobic relevant measures and major findings of the reviewed studies

Author	Relevant Measures	Major Findings
Almeida et al. 2020 [1]	MAV, V̇O_2_peak, La, ΔLa	Absolute V̇O_2_peak and MAV were significantly correlated with swimmers performance at PB50 (*r* = − 0.81, *r* = − 0.70, *P* < 0.01), PB100 (*r* = − 0.82, *r* = − 0.77, *P* < 0.01) and PB200 (*r* = − 0.75 and *r* = − 0.75, *P* < 0.01), respectively. VO_2_peak of each maximal test was correlated with the swimmers personal best times (*r* = -0.82, -0.84, -0.76, *P* < 0.01, for the 50, 100 and 200 m tests, respectively)
Chatard et al. 1990 [9]	V̇O_2_max	V̇O_2_max and 400 m freestyle time positively correlated in males (*r* = 0.70; *p* < 0.01) and females (*r* = 0.72; *p* < 0.01)
de Barros Sousa et al. 2017 [12]	Fmax, tFmax, Fmean, Fmin, FI, SLOPE, LMI iTSLacmin	100 m freestyle time negatively correlated with iTSLacmin (*r* = -0.67; *p* = 0.04), Fmax (*r* = -0.79; *p* < 0.01), tFmax (*r* = -0.68; *p *= 0.03), Fmean (*r* = -0.72; *p* = 0.02), Fmin (*r* = -0.67; *p* = 0.03) and positively with SLOPE (*r* = 0.82; *p* < 0.01). 200 m freestyle time negatively correlated with iTSLacmin (*r* = -0.80; *p* < 0.01), Fmax (*r* = -0.91; *p* < 0.01), tFmax (*r* = -0.63; *p* = 0.05), Fmean (*r* = -0.87; *p* < 0.01), Fmin (*r* = -0.84; *p* < 0.01) and positively with SLOPE (*r* = 0.87; *p* < 0.01). No significant correlations were found between 100 and 200 m freestyle performance and FI
de Mello Vitor & Böhme, 2010 [13]	AnP, CS	100 m freestyle average speed positively correlated with AnP (R2 = 0.67; *p* < 0.01) and CS (R2 = 0.34; *p* < 0.01)
Duché et al. 1993 [16]	V̇O_2_max, MP	V̇O_2_max positively correlated with 50 m (*r* = 0.70; *p* < 0.01) and 400 m freestyle performance (*r* = 0.67; *p* < 0.001). MP in 30 s cycle ergometer positively correlated with 100 m (*r* = 0.59; *p* < 0.05) and 400 m (*r* = 0.42; *p* < 0.05) freestyle performance
Espada et al. 2015 [17]	V̇O_2_max, v V̇O_2_max, MAV, 97.5% (infra) and 102.5% (supra) MLSSv	vV̇O_2_max negatively correlated with 400 m (*r* = -0.70; *p* < 0.01) and 800 m (*r* = -0.72, *p* < 0.01) freestyle time. 400 m freestyle time positively correlated with time constant at Infra-MLSSv (*r* = 0.64; *p* < 0.03). 800 m freestyle performance positively correlated with time constant at infra-MLSSv (*r* = 0.75; *p* < 0.01) and supra-MLSSv (*r* = 0.58; *p* ≤ 0.05)
Ferreira et al. 2021 [21]	BLc, ΔLa	ΔLa positively correlated with 400 m freestyle speed improvements (*r* = 0.35; *p* < 0.05). BLc positively correlated with 400 m performance at four testing moments in the season (*r* = 0.50, 0.72, 0.62, 0.55, *p* < 0.05)
Hawley et al. 1992 [29]	AnP, peak sustained workload	Relationship between 50 m freestyle speed and MP of arms (*r* = 0.63) and legs (*r* = 0.76) Relationship between 400 m speed and peak sustained workload (*r* = 0.70)
Hellard et al. 2018 [31]	V̇O_2_, BL, total energy expenditure, aerobic, alactic and lactic anerobic contributions	Faster 100 m freestyle performance associated with higher V̇O_2_ and aerobic power
Jürimäe et al. 2007 [35]	V̇O_2_peak, FFM, Cs, ΔLa	400 m freestyle time negatively correlated with V̇O_2_peak (*r* = -0.618; *p* = 0.0001) and FFM (*r* = –0.593; *p* < 0.05). BF%, ΔLa and Cs had no relationship with 400 m freestyle performance
Kalva-Filho et al. 2018 [39]	LMI, AnP	LMI positively correlated with 200 m freestyle swimming speed (*r* = 0.71; *p* = 0.001) and 30 min freestyle swimming speed (*r* = 0.70; *p* = 0.004). MF positively correlated with 200 m freestyle speed (*r* = 0.82; *p* = 0.001) and 30 min freestyle speed (*r* = 0.76; *p* = 0.001)
Klika & Thorland, 1994 [41]	Peak V̇O_2_, arm-stroke force and leg stroke force	Multiple discriminant function identified leg kick force, peak V̇O_2_, stroke efficiency and muscularity as predictors of performance level (Multiple discriminant function coefficient = unstandardized 0.822, 0.221, 0.732; standardized 1.87, 1.48, 1.08
Lätt et al. 2009 [42]	Cs, V̇O_2_, ∆La	V̇O_2_ (R2 > 0.346; *p* < 0.05) predicted 400 m freestyle swimming performance. Stepwise regression analysis revealed all bioenergetical factors combined (∆La, Cs and predicted VO_2_) predicted 400 m freestyle performance (R2 > 0.311; *p* < 0.05)
Lätt et al. 2010 [43]	Cs, V̇O_2_peak, ∆La	∆La and Cs negatively correlated with 100 m freestyle performance (*r* = -0.598; -0.544; P ≤ 0.05). Multiple linear regression identified ∆La, La3, La5 and Cs positively correlated with 100 m swimming performance (R2 = 0.551; *p* = 0.004). V̇O_2_ parameters were not significantly correlated with swimming performance
Maszczyk et al. 2012 [50]	Lung capacity	Lung capacity were key predictive factors of 50 m freestyle performance in when used in regression models
Mezzaroba et al. 2013a [51]	LM, BF%	LM correlated with 100 m, 200, and 400 m freestyle in males (*r* = 0.92, 0.97, 0.96, *P* < 0.001) and females (*r* = 0.89, 0.91, 0.80, *P* < 0.001) respectively. LM did not correlate with BF%
Mezzaroba et al. 2013b [52]	LM, Lapeak,CS	LM and CS predicted 100 m, 200 m and 400 m freestyle times in males (r2 = 0.951, 0.992, 0.988) and females (r2 = 0.816, 0.950, 0.992), respectively
Mitchell et al. 2018 [53]	CS	CS indicated as a performance indicator for male and female 200 m swimmers (*r* = -0.42; -0.47) through multiple linear regression model
Morouço et al. 2014 [58]	AnP, MAV, MAI	50 m freestyle swimming speed positively correlated with MAV (*r* = 0.76; 0.81; *p* < 0.001) and MAI (*r* = 0.91; 0.70; *p* < 0.001)
Papoti et al. 2009 [61]	AC, MLSS	AC significantly correlated with 400 m freestyle velocity
Papoti et al. 2013 [60]	V̇O_2_max, LT, CF, AnIMPc, AnF	iV̇O_2_max, LT, CF, AnIMPc and AnF positively correlated with 100 m freestyle (*r* = 0.89; 0.70; 0.48; 0.76; 0.86; *p* < 0.05) 200 m freestyle (*r* = 0.89; 0.74; 0.63; 0.66; 0.78; *p* < 0.05) and 400 m freestyle performance (*r* = 0.92; 0.80; 0.60; 0.59; 0.71; *p* < 0.05)
Pardos-Mainer et al. 2015 [62]	V̇O_2_max	V̇O_2_max had significant correlations with 50 m freestyle time (*r* = -0.435; *p* < 0.05)
Reis et al. 2010 [71]	V̇O_2_, BL, BF%	200 m breaststroke performance was predicted by the combination of aerobic fraction on energy release (AER), peak BL post-exercise and VO_2_ elicited at the swimming velocity corresponding to the 2 mmol.L-1 threshold. 100 m breaststroke performance was predicted by the combination of BF%, V̇O_2_ elicited at the swimming velocity corresponding to the 4 mmol.L-1 threshold and VO_2_peak
Ribeiro et al. 1990 [72]	V̇O_2_max, lactate max	400 m freestyle performance positively correlated with v85%VO_2_max (*r* = 0.90; *p* < 0.01) and vBL4mmol (*r* = 0.89; *p* < 0.01). Multiple linear regression analysis found the v85% V̇O_2_max and the vBL4mmol positively correlated with maximal swimming velocity in 400 m freestyle (R2 = 0.83, *p* < 0.001)
Rodríguez et al. 2015 [74]	Lapeak	100 m freestyle mean swimming speed positively correlated with Lapeak (*r* = 0.73; *p* = 0.0001)
Saavedra et al. 2010 [76]	Aerobic endurance, speed endurance	Positive correlations between swimming performance and endurance shuttle run (*r* = 0.369; p ≤ 0.05), 30 min test (*r* = 0.700; p ≤ 0.05), 6 × 50 speed endurance (*r* = 0.685; p ≤ 0.05)
Smith et al. 1988 [81]	V̇O_2_, V̇O_2_max	100 m and 200 m backstroke times were negatively correlated with WA-VO_2_ (*r* = -0.50; -0.66). V̇O_2_max was positively correlated with 100 m backstroke velocity (*r* = 0.74) and 200 m backstroke velocity (*r* = 0.48)
Strzala et al. 2015 [82]	V̇O_2_peak	200 m breaststroke velocity positively correlated with V̇O_2_peak (*r* = 0.41; *p* < 0.05), 200 m turning performance positively correlated V̇O_2_peak (*r* = 0.41; *p* < 0.05)
Unnithan et al. 2009 [86]	V̇O_2_, Cs	V̇O_2_ relative to BW was positively correlated with national ranking at 200 m (*r* = 0.67; *p* < 0.05). Cs at 1.1 m · s − 1 negatively correlated with national ranking at 50 m (*r* = -0.66; *p* = 0.038), 100 m (*r* = -0.83; *p* = 0.003), 200 m (*r* = -0.73; *p* = 0.017), 500 m (*r* = -0.811; *p* = 0.004) and 1000 m (*r* = -0.678; *p* = 0.031) and positively for race time at 200 m (*r* = 0.783; *p* = 0.007)

Tests relating to anaerobic determinants of swimming performance were used in eight studies. Tethered swimming performance over 30 s [12, 58, 61] and 22.9 m [41] showed moderate to very strong correlations with swimming performance. Papoti et al. [60], also found moderate to strong correlations between 100 m, 200 m and 400 m freestyle performance with anaerobic impulse capacity and critical force over four short, tethered swimming bouts. Tests using ergometers to assess anaerobic measures were conducted for the upper [29] and lower body [16, 29], where measures of force, power and fatigue were associated with swim performance. Anaerobic power was also measured using average velocity in an 8 × 25 m all out swimming test which showed moderate correlations with 100 m freestyle performance [13]. In one study, speed endurance during a specific swimming test was reported to have a moderate correlation with LEN scores [76]. Pardos-Mainer et al. [62] presented a moderate correlation between 30 m sprint running velocity and 50 m freestyle time.

BL profiles were measured across 13 studies, which used tethered [12, 36, 60, 61] and free-swimming tests [17, 21, 42, 43, 51, 61, 71, 72, 74] to assess these parameters. Net change in BL concentration was analysed in relation to swim performance in two investigations, one found a moderate correlation with 100 m freestyle performance [43] and one did not report it was a successful predictor of performance [42]. Three studies measured BL concentration after a single maximal effort bout of swimming, one found no relationship [72], the other two found weak to strong correlations with performance improvements over time [21] and mean swimming speed [74]. Ribeiro et al. [72] found a strong correlation between velocity at BL 4 mmol and maximal swimming velocity. One study identified relationships between infra and supra intensities of maximal lactate steady state with 800 m freestyle and 400 m freestyle performance at infra intensities only [17]. Lactate threshold was measured by Papoti et al. [60], who found strong correlations with swim performance across multiple distances. Lactate minimum tests and its related parameters were associated with swim performance in four studies [12, 36, 51, 52].

Measurements of V̇O_2_ were observed in 17 studies [1, 9, 16, 17, 31, 35, 41–43, 60–62, 71, 72, 81, 82, 86]. V̇O_2_peak was measured in seven studies, four of which showed weak to strong relationships with swimming performance [1, 35, 42, 82]. One analysis showed V̇O_2_peak was a contributor to swim performance when entered into a multi-discriminant function with leg kick force, stroke efficiency and muscularity [41]. Two studies found no relationships between V̇O_2_peak and swimming performance [43, 86]. Measures of V̇O_2_max showed moderate to very strong relationships with swimming performance in seven studies [9, 16, 17, 60, 62, 72, 81]. One investigation measured aerobic capacity via a staged shuttle run and 30-min swim test, where weak and strong correlations were found between tests and LEN scores [76]. Another study measuring aerobic capacity through swimming tests found that 400 m freestyle velocity and maximal lactate steady state (MLSS) were correlated to this measure [61]. One study found that measures of V̇O_2_ and aerobic power were associated with faster 100 m freestyle performance [31]. Three studies investigated critical speed, a measure of aerobic threshold, finding weak and moderate correlations with swimming performance [10, 46, 52]. One study measured lung capacity which was found to be a predictive factor of 50 m freestyle performance when used in regression models [50]. Breaststroke performance for the 100 m and 200 m events was successfully predicted by combinations of BL and V̇O_2_ in a study evaluating breaststroke performance measures [71].

The energy cost of swimming, which considers anaerobic and aerobic components of swimming performance, was measured in four studies. Relationships were reported in two investigations that found links between energy cost, 100 m freestyle performance [43] and national ranking over multiple distances [86]. The other studies did not report performance links but did show relationships between energy cost and maturation stage [35, 42].

### Body composition measures

Out of the 18 studies that investigated body composition, seven found some relationship with swimming performance (Table 4). Six studies found weak to very strong relationships between BF% [5, 15, 62, 71, 76, 78] and swim performance, however, each did not identify BF% as a predictive factor. Saavedra et al. [76], identified a weak correlation between swimming performance and lower BF% in males, but no association in females. Seffrin et al. [78], found higher BF% was very strongly associated with faster swim times in females, but had no association in males. Klika and Thorland [41], identified greater fat mass was associated with faster sprint swimming times. Mitchell et al. [53], found 100 m freestyle and 200 m freestyle swimmers had significantly different BF% (62.9 vs. 68.9, *p* < 0.01). One study found that faster swimmers could be categorised by BF%, where faster swimmers had overall lower sum of skinfolds than slower swimmers [5]. Six studies identified LBM [52, 78, 82, 83] and fat free mass [35, 42, 62] as having weak to very strong relationships, where higher levels were beneficial to performance. Pardos-Mainer et al. [62] did not report fat free mass was a predictive value, although it showed a moderate correlation with swimming performance. Other investigations found no significant relationships with body composition measures and swim performance, including BF% [13, 16, 25, 43, 51], LBM [41] and fat free mass [43].

**Table 4:** Summary of body composition relevant measures and major findings of the reviewed studies

Author	Relevant Measures	Major Findings
Dos Santos et al. 2021 [15]	BF%	BF% amongst best predictors of 50 m freestyle performance in backwards regression analysis, where lower BF% conducive to performance
Bond et al. 2015 [5]	Skinfolds	Swimmers who were catagorised as "fast swimmers" had overall lower sum of skin folds than "slow swimmers" and correlated with 100 m freestyle time (*r* = 0.410, *P* < 0.01)
de Mello Vitor & Böhme, 2010 [13]	BF%	BF% showed no relationship with performance
Duché et al. 1993 [16]	BF%	No significant between body fat percentage and freestyle performance
Geladas et al. 2005 [25]	BF%	No correlations between BF% and 100 m freestyle time
Jürimäe et al. 2007 [35]	FFM, BF%	400 m freestyle time negatively correlated FFM (*r* = –0.593; *p* < 0.05). BF% had no relationship with 400 m freestyle performance
Klika & Thorland, 1994 [41]	FM, LBM	91.4 m freestyle performance positively correlated with FM (*r* = 0.61; *p* < 0.05), not LBM
Lätt et al. 2009 [42]	FFM	FFM predicted 400 m freestyle performance in multiple regression analysis (R2 > 0.318; *p* < 0.05)
Lätt et al. 2010 [43]	BF%, FFM	BF% and FFM were not significantly correlated with swimming performance
Mezzaroba et al. 2013b [51]	LBM, FM	LBM predicted 100 m, 200 m and 400 m freestyle time in males (r2 = 0.784, 0.853, 0.743). LBM and FM predicted 100 m, 200 m and 400 m times in females (r2 = 0.524, 0.439, 0.357), respectivley
Pardos-Mainer et al. 2015 [62]	BF%, FFM	BF% and FFM had significant correlations with 50 m freestyle time (*r* = -0.316; -0.516; *p* < 0.05)
Saavedra et al. 2010 [76]	BF%	Positive correlations between swimming performance and BF% (*r* = 0.259; p ≤ 0.05),
Seffrin et al. 2021 [78]	LBM, BF%	LBM negatively correlated with 100 m freestyle performance in males (*r* = -0.60; -0.83; p ≤ 0.05). BF% positively correlated with 100 m (*r* = 0.84; 0.85; p ≤ 0.05) and 400 m freestyle performance (*r* = 0.97; p ≤ 0.05) in females
Strzala et al. 2015 [82]	LBM	200 m breaststroke turning performance positively correlated with LBM (*r* = 0.38; *p* < 0.05)
Strzała et al. 2019 [83]	LBM	Positive correlation between LBM and freestyle velocity (*r* = 0.78; *p* < 0.01)

## Discussion

This systematic review aimed to evaluate studies which investigated physical determinants of swimming performance in dryland exercises in youth swimmers. To our knowledge, no review currently exists on this subject. Although there are some inconsistencies within the reviewed literature, these can be put down to differences in methodologies and characteristics of study participants. Studies in this review scored well in the JBI assessment, with no cross-sectional study scoring under four (out of eight) and no randomised-controlled trial scoring under eight (out of 13). Our review suggests greater anaerobic and aerobic capabilities, maximal strength, explosive power and LBM are related to superior swim performance in youth swimmers, with BF% being a more nuanced variable. Therefore, training prescriptions may be better informed after considering this review.

### Strength and power

Maximal strength has a well-documented relationship with sub-maximal strength, where repetition performance at sub-maximal loads correlates with 1RM [34]. Keiner et al. [38] and Keiner et al. [39] identified 1RM scores as having relationships with sprint swim performance in various upper and lower body movements from 5 to 100 m, suggesting ability to produce force is an important factor. Force generated through the arm stroke is reported to contribute to swimming velocity and performance [12]. Since the arm stroke in swimming is submaximal in terms of load resistance, it could be hypothesised improving upper body maximal strength may be a useful tool for coaches to enhance swimming performance. Isokinetic and hand grip strength measures were similar to the aforementioned results, where higher force output related to better swim performance [23, 62, 76, 78, 83], further implying general overall strength qualities are important in swimming. On the other hand, some studies found isometric strength measurements of multi-joint [44] and single-joint [23] movements do not relate to swimming or start performance suggesting dynamic strength is a more important quality due to the nature of the muscle contractions involved in swimming.

The CMJ and SJ are very similar in biomechanics to the start and turn in swimming, reasonably improving vertical jump ability would enhance start and turn performance according to the dynamic correspondence principle [87]. This is supported by Potdevin et al. [70], where the intervention group improved maximal glide speed after 6 weeks of plyometric training. This study identified changes in squat jump height related to changes in 50 m freestyle velocity that included a dive start, similar to Mitchell et al. [53] for 100 m freestyle. Although actual start performance to 15 m was not measured, the start accounts for around 30% of 50 m freestyle and 15% of 100 m freestyle time [46], suggesting a connection between these measures. Loaded jump performance was related to swimming start performance in two studies [23, 53], where moderate to strong correlations (*r* = 0.40–0.79) were found throughout, regardless of load or distance. García-Ramos et al. [23] reported loaded vertical jumps had stronger correlations than unloaded vertical jumps with starts, suggesting lower body speed-strength is an important component of swimming start performance. One study included in this review did not identify relationships between jumping and swim performance [41], but as previously indicated, the type of jump was not clearly stated and start or turn performance was not specifically measured, reducing the validity of this finding.

### Anaerobic and aerobic

The influence of anaerobic capacity components on swim performance are described in this review, where they seem to play an influential role in determining performance of youth swimmers. Tests for maximal sprinting capabilities were conducted in four studies [12, 41, 58, 60]. These tests are designed to simulate the physiological responses of sprint swimming whilst having the ability to measure force parameters. Each study found higher maximal or average stroke forces were associated with superior swim performance. Morouço et al. [58] identified maximum force in swimming had a non-linear relationship with sprint swimming speed, implying a limit in force to enhance swimming speed inevitably occurs. Impulse force was considered to be a greater indicator of performance, as force time characteristics are a better reflection of stroking mechanics at high intensities. Non-swimming tests conducted using ergometers supported this notion, where mean power rather than maximal power of the upper and lower limbs was identified as a better indicator of sprint and middle distance freestyle swimming [16, 29]. Potential contributors to anaerobic power, and consequently swim performance, may include body mass, height, hand width and biacromial breadth [13].

Acute increases in BL concentration are associated with reductions in performance, as lactate-induced acidosis disturbs the cross-bridge cycle, impairing contractile ability of muscle cells [14]. Nevertheless, higher BL concentrations post exercise are associated with higher swimming velocities [43, 72, 74], suggesting ability to produce lactate relates to faster swimming. This is further supported by Ferreira et al. [21] who identified changes in post exercise BL concentration over time increased as 400 m freestyle performance improved. One study contradicted these findings, as changes in BL concentration over time had no association with improvements in swim performance [42]. Lactate threshold is a measurement that provides insight to the aerobic capabilities of a swimmer, where raising lactate threshold is associated with the ability to work at a higher rate at the same BL concentration. Findings in this review explain velocity or intensity associated with lactate threshold is an important indicator of swim performance, where capacity to maintain a higher swimming speed at lactate threshold determines swimming performance [12, 36, 60, 72].

Oxygen uptake and its associated measures are considered as one of the most important factors in swimming success, especially for middle and long distance events where aerobic contribution can reach up to 90% [73]. V̇O_2_peak is directly related to V̇O_2_max, where the highest value of V̇O_2_ is recorded. In this review, results are mixed as three studies demonstrated higher V̇O_2_peak values partially determines swimming times in 200 m and 400 m events [35, 42, 81] and two studies found no association [43, 85]. A rationale for differences in results may be the influence of confounding factors that have associations with V̇O_2_peak. To exemplify, Pendergast et al. [64] explained V̇O_2_peak may not associate with race times is due to high variability in energy cost between swimmers, where multiple factors such as anaerobic power and stroke mechanics play a crucial role. Overall, V̇O_2_max scores seem to be a more reliable determinant of swimming performance where all studies that measured it found significant relationships with swimming performance [9, 16, 17, 60, 62, 72, 81]. V̇O_2_max alone accounted for 50% of variance in 400 m freestyle performance in one study [9], demonstrating its important role in middle-distance performance. Generally, the intensity or velocity associated with V̇O_2_max is a better predictor of performance, as this measure takes exercise economy into account, making it more specific to actual swim performance rather than pure energetic capabilities [17, 60]. Similarly to V̇O_2_peak, V̇O_2_max is influenced by confounding variables, highlighted by Duché et al. [16], who noted V̇O_2_max was not a predictor of performance when height was added into the analysis.

Critical speed is a measure of aerobic swimming threshold, considering energetic capabilities and stroke efficiency. Mitchell et al. [53] reported 200 m specialists tended to have higher critical speed values than 100 m specialists, indicating aerobic capacity is more important in longer events, even though it was considered determining factor of 100 m performance by de Mello Vitor and Böhme [13]. Energy contribution in 100 m freestyle is considered to be 55% anaerobic and 45% aerobic [69], versus 200 m freestyle which is deemed 35% anaerobic and 65% aerobic [85], further explaining the differences in importance of aerobic capacity across events.

### Body composition

Possessing a higher BF% has been suggested to present benefits to buoyancy, thus enhancing swimming performance [45]. On the other hand, high BF% means larger body surface area, increasing drag forces the swimmer attains [10]. Klika and Thorland [41] supported the idea that higher BF% is conducive to swimming performance, where faster swimming velocities were related to a higher BF%. The results from Seffrin et al. [78] were akin in females, where very strong relationships were found between higher BF% and swim performance, contrary to Saavedra et al. [76] who found males had the opposite relationship and females showed no association with swimming performance. One study found that swimmers categorised as “fast” had a lower overall sum of skin folds [5] and another finding that lower BF% was a predictor of 50 m freestyle performance when used in a backwards regression analysis [15]. One other study found some relationships between BF% and anaerobic measures of swimming performance [71]. All other studies found no associations with BF% and swim performance, suggesting females may benefit from higher BF%, but the outcome for males is unclear. Similar to BF%, gross increases in LBM may hamper swimming performance due to greater body surface area, increasing drag forces applied to the swimmer [10]. However, results of this review suggest greater LBM is associated with superior swimming performance [35, 52, 62, 77, 81–83]. Although this may seem confounding, the aforementioned relationships between strength and swim performance may be linked to measures of LBM, as muscle cross-sectional area is associated with strength capabilities [49]. Therefore, increasing muscle mass of the force generating muscles in swimming may be a worthwhile strategy to enhance swimming performance, outweighing detriments associated with increased body surface area.

### Multivariate analysis

As swimming performance is not binary in its determining elements, unsurprisingly some studies identified a combination of factors that best predicted swimming performance. This is emphasised in one study that identified 78% of variance in swim performance between subjects was determined by the combination of leg kick force, V̇O_2_peak, stroke efficiency and muscularity [41]. Furthermore, Lätt et al. [43] found biomechanical factors may explain 90.3% of variance in 100 m swim performance compared to anthropometrical (45.8%) and physiological (45.2%) parameters. Likewise, Lätt et al. [42] observed a combination of biomechanical factors (R^2^ > 0.322; *p* < 0.05) better characterised 400 m swimming performance compared to bioenergetic (R^2^ > 0.311; *p* < 0.05) and physical factors (R^2^ > 0.203; *p* < 0.05). These findings suggest biomechanical factors are better at predicting performance than physiological and anthropometric measurements even though the latter two still were still considered valid determinants, illustrating swimming performance is a multi-factor variable. Within the physiological variables, the combination of horizontal jump and lung capacity have been used to predict sprint freestyle performance in regression models [50], demonstrating the importance of metabolic and power components of performance.

### Limitations and future research

A large portion of studies did not include training interventions, or study their subjects over time, meaning performance improvements of selected variables were not accounted for. This means although swimming performance may be related to a particular parameter at a certain time point, it cannot be confirmed improving any physiological capacity will directly influence performance. Furthermore, although a relatively substantial number of studies were included in this review, important parameters including maximal strength and BF% were only measured in a small number of studies, increasing the chance of false conclusions being drawn. Finally, many studies presented a shortage of detail when describing confounding variables, (e.g., stroke preference, event specialisation and strength training experience), meaning the influence of physiological capacities on performance may have been affected by hidden variables.

To the benefit of dryland practices, randomised-controlled trials should focus on strength and power training interventions that measure changes with swimming performance over a period of time. Particularly, examining differences between strength and power training in sprint, middle and long distance swimmers across strokes. Investigating the mechanisms between body composition and swimming performance would be a worthwhile area of study, as the current research is conflicting. Moreover, determining optimal levels of LBM and BF% in relation event distance, stroke and performance should be considered as a valuable area of examination.

## Conclusion

This review highlights that various physical characteristics contribute to improved swimming performance in youth athletes. Superior strength, power, LBM, anaerobic and aerobic qualities are important factors. However, the relationship between BF% and swimming performance is uncertain. Coaches should prioritize general strength and power training, along with anaerobic and aerobic training, to enhance performance. Strength seems to be beneficial for actual swimming speed, where are power has strong relationships with start and turn performance. Anaerobic power is particularly important for maximal effort sprinting, while aerobic capabilities play a bigger role in longer events. The ability to produce more BL is associated with faster swimming times. Both genders benefit from higher LBM, likely due to its association with strength and power. Manipulating BF% in females should be done with caution due to inconclusive findings. This review provides a better understanding of youth swimming performance and dryland assessments, suggesting youth swim performance is influenced by a combination of physiological markers.

## Data Availability

Data sharing is not applicable to this article as no datasets were generated or analysed during the current study.

## Abbreviations

1RM: 1-Repetition maximum
AF: Average force
AnF: Anaerobic fitness
AnIMPc: Anaerobic impulse capacity
AnP: Anaerobic power
BF%: Body fat percentage
BJ: Broad jump
BL: Blood lactate
BLc: Blood lactate concentration
BW: Body weight
CF: Critical force
CMJ: Countermovement jump
CS: Critical speed
Cs: Energy cost of swimming
EPF: Estimated propulsive force
FFM: Fat free mass
FI: Fatigue index
FM: Fat mass
Fmax: Maximum force
Fmean: Mean force
Fmin: Minimum force
HGS: Hand grip strength
HJ: Horizontal jump
IMP: Impulse
iTSLacmin: Lactate minimum intensity
i V̇O_2_max: Intensity at V̇O_2_ max
JBI: Joanna Briggs Institute
Lapeak: Lactate peak
LBM: Lean body mass
LMI: Lactate minimum intensity
LT: Lactate threshold
MAI: Maximum average impulse
MAV: Maximal aerobic velocity
MLSSv: Velocity associated with maximal lactate steady state
MP: Mean power
MPP: Mean propulsive power
PFA: Propulsive force of arms
PP: Peak power
RFD: Rate of force development
SJ: Squat jump
SLOPE: Inclination slope from maximum to minimum force
tFmax: Time to maximum force
TOV: Take off velocity
TV: Throwing velocity
vBL: Velocity at blood lactate concentration
Vcrit: Critical velocity
v V̇O_2_: Maximum velocity associated with V̇O_2_ max
WA- V̇O_2_: Weight adjusted V̇O_2_
ΔLa: Net change in blood lactate concentration
